# The association between supportive social ties and autonomic nervous system function—differences between family ties and friendship ties in a cohort of older adults

**DOI:** 10.1007/s10433-021-00638-2

**Published:** 2021-07-09

**Authors:** Catherin Bosle, Hermann Brenner, Joachim E. Fischer, Marc N. Jarczok, Ben Schöttker, Laura Perna, Kristina Hoffmann, Raphael M. Herr

**Affiliations:** 1 Mannheim Institute of Public Health, Social and Preventive Medicine, Mannheim Medical Faculty, Heidelberg University, Mannheim, Germany; 2 Division of Clinical Epidemiology and Aging Research, German Cancer Research Center (DKFZ), Heidelberg, Germany; 3 Network Aging Research, University of Heidelberg, Heidelberg, Germany; 4 Department of Psychosomatic Medicine and Psychotherapy, Ulm University Medical Center, Ulm, Germany

**Keywords:** ANS function, Family ties, Friendship ties, Heart rate variability, Older adults, Social ties

## Abstract

Supportive family and friendship ties can serve different functions and thus might show different associations with an individual’s health. Particularly, older adults might show varying health benefits of different types of supportive ties depending on their marital and retirement status. Our aim is to analyze relationships between different types of supportive social ties and autonomic nervous system (ANS) function, a physiological indicator of health that can help to establish the biological plausibility of the association—measured by heart rate variability (HRV). We present cross-sectional linear regression analyses of a German cohort of community-dwelling older adults (2008–2010; *n* = 1,548; mean age = 68.7 years). Our findings indicate that supportive friendship ties show significant positive associations (i.e., higher HRV) in individuals that are either not married or above retirement age. Supportive family ties show significant positive associations in individuals below retirement age. Significant results vanish or are reduced after accounting for behavioral/physical and psychological/cognitive indicators. We conclude that programs supporting the development or maintenance of friendship ties might be especially beneficial in unmarried older adults and adults above retirement age.

**Supplementary Information:**

The online version contains supplementary material available at 10.1007/s10433-021-00638-2.

## Background

Over the past decades, a large amount of research has examined the relationship between social ties and measures of both morbidity and mortality. Having fewer social ties has been associated, for example, with decreased subjective health (Thanakwang 2009), a higher prevalence of depression (Han et al. 2019), and both increased all-cause and cause-specific mortality (Holt-Lunstad et al. 2010; Shor et al. 2013). Additionally, in their meta-analysis, Holt-Lunstad et al. (2010) suggested that the association between social ties and mortality is similar in magnitude to other well-established risk factors for mortality, such as tobacco use, alcohol abuse, obesity, and physical inactivity.

A high number of social ties, especially supportive ones, are thought to be particularly beneficial to the overall health and well-being of older adults (Lubben and Gironda 2003), who are more likely to face physical and mental health decline, including diagnoses of multiple chronic diseases (WHO 2015). Additionally, older adults are likely to experience major life events, such as retirement or widowhood, resulting in significant changes to the quantity and quality of their social ties (Cornwell et al. 2008).

Social ties are defined “as a construct consist[ing] of various features of social connections between an individual and the members of various primary and secondary social groups with whom the individual is involved” (Lubben and Gironda 2003, page 321–322). Research on supportive social ties has often focused on the contribution of the spouse (Shor et al. 2013). While a spouse represents one of the closest social ties, further exploration of other supportive ties and their effects on health may be valuable, especially when individuals do not have a spouse. Other family members, friends, and neighbors also represent the primary social groups of individuals (Lubben and Gironda 2003). However, as family ties and friendship ties can serve different functions, they may affect the individual differently. Both family and friendship ties are thought to provide emotional support (Huxhold et al. 2013; Messeri et al. 1993). Friends and spouses are similar in that they both, for example, provide companionship, offer opportunities for social integration, and may foster a greater sense of self-worth (Huxhold et al. 2013; Messeri et al. 1993). Additionally, friendship ties may be especially important, as friends are often chosen according to similarities in interests or experiences (Thanakwang 2009; Zunzunegui et al. 2004). Compared to family ties, friendship ties can be more easily terminated if they do not fulfill expectations or become adverse and those that persist into old age might therefore be especially close and supportive (Birditt et al. 2009). Friendship ties may require more maintenance, but they are also expected to be more reciprocal (Blieszner and Roberto 2004). On the other hand, due to their proximity and function family ties may be crucial when it comes to providing instrumental support (e.g., financial aid, physical care), especially in the case of illness or functional decline (Messeri et al. 1993). However, in contrast to other types of support, instrumental support may lead to emotional distress and feelings of vulnerability, thus diminishing the positive effects of support (Li and Zhang 2015).

Findings on the association between different social ties and both morbidity and mortality are mixed. For example, one study found positive associations between friendship ties and physical health, but none for family ties (Thanakwang 2009). On the other hand, a meta-analysis reported significant associations between mortality and family ties and no associations between mortality and friendship ties (Shor et al. 2013). Similarly, it was found that the level of C-reactive protein, a physiological marker of inflammation, was negatively associated with the number of supportive family ties but not with the number of supportive friendship ties (Uchino et al. 2015).

The beneficial effects of different supportive social ties may be dependent on various life circumstances an older adult might have experienced. Changes in marital status (e.g., divorce, widowhood) and retirement are life transitions that can further lead to significant changes in one’s needs and social ties. The absence or loss of a spouse may be characterized by significantly fewer social ties (Cornwell et al. 2008). The hierarchical compensatory model assumes that sources of social support are approached by ordering preferences: (1) spouses, (2) other family members, (3) friends or neighbors (Cantor 1979). While the task-specific model also assumes an ordering preference, it states, more specifically, that individuals request support from ties that best fulfill the requirements for the task at hand and that the spouse is most often the closest and most capable tie (Litwak 1985; Messeri et al. 1993). Both models suggest that, depending on the presence of a spouse, the importance of other social ties might vary. One study analyzing support from friends and family and emotional well-being in elderly found more support for the task-specific model than the hierarchical compensatory model (Li et al. 2014).

Retirement, on the other hand, is thought to be associated with the reduction of work-related ties (Cornwell et al. 2008). Moreover, socio-emotional selectivity theory implies that due to the limited remaining lifetime, only social ties evaluated as emotionally meaningful are kept (Carstensen et al. 1999). Similarly, the strength and vulnerability model (Charles 2010) argues that negative ties are avoided later in life as older adults, given their life experience, can better strategize how to minimize negative emotional encounters.

As recent research on the association between supportive family and friendship ties and measures of morbidity and mortality indicates mixed findings, we chose to explore the association with more immediate health endpoints that could help in establishing its biological plausibility (Thoits 2011; Uchino 2006). Several socio-demographic characteristics are already known to be associated with ANS function such as gender, age, marital status, and living arrangements (Abhishekh et al. 2013; Randall et al. 2009). Biological mechanisms have long been suggested as one of the pathways to explain the relationship between social ties and health (Berkman and Glass 2000; Uchino 2006). Based on the model proposed by Uchino (2006), supportive social ties affect behavioral and psychological processes, which, in turn, impact biological processes such as cardiovascular, neuroendocrine, and immune function (for systematic reviews on the association between social ties and biological processes, see Ditzen and Heinrichs (2007); Uchino (2006)).

Autonomic nervous system (ANS) function, measured through heart rate variability (HRV), may be a particularly important physiological pathway through which supportive social ties affect health. Regulated ANS function, as indicated by greater HRV, is thought to be a useful indicator of healthy heart function (Thayer et al. 2012) and has been positively associated with higher self-rated health (Jarczok 2015). Conversely, dysregulated ANS function, as reflected by low HRV, is associated with cardiovascular disease risk factors such as hypertension and diabetes (Thayer et al. 2010).

Although initially assessed in patient samples with coronary diseases, HRV has been increasingly used in research on health in the general population (Britton et al. 2007). Both animal and human studies have shown that (supportive) social ties are related to ANS function in that the lack of social ties is negatively correlated with HRV (Gouin et al. 2015; Grippo 2011; Hemingway et al. 2005; Horsten et al. 1999). Further studies have shown significant positive associations with both marital status and marital quality (Randall et al. 2009; Smith et al. 2011). Findings in this area are not uniform, however, as Britton et al. (2007) found no significant associations between social ties and several HRV measures.

The aim of the present exploratory study is to analyze the cross-sectional relationship between supportive social ties and several indicators of ANS function in terms of HRV in a cohort of older adults. To add to previous work, this study focuses on differences in this association by accounting for the type of supportive social tie—either family ties or friendship ties. We also explore the extent to which these associations change when differentiating between marital status and retirement status, two types of life circumstances that are likely to change with age and can alter available supportive social ties. This approach was chosen, as the knowledge about how different social ties affect individuals in different life circumstances can be important in generating targeted interventions.

## Data and methods

### Study population

**Fig. 1:**
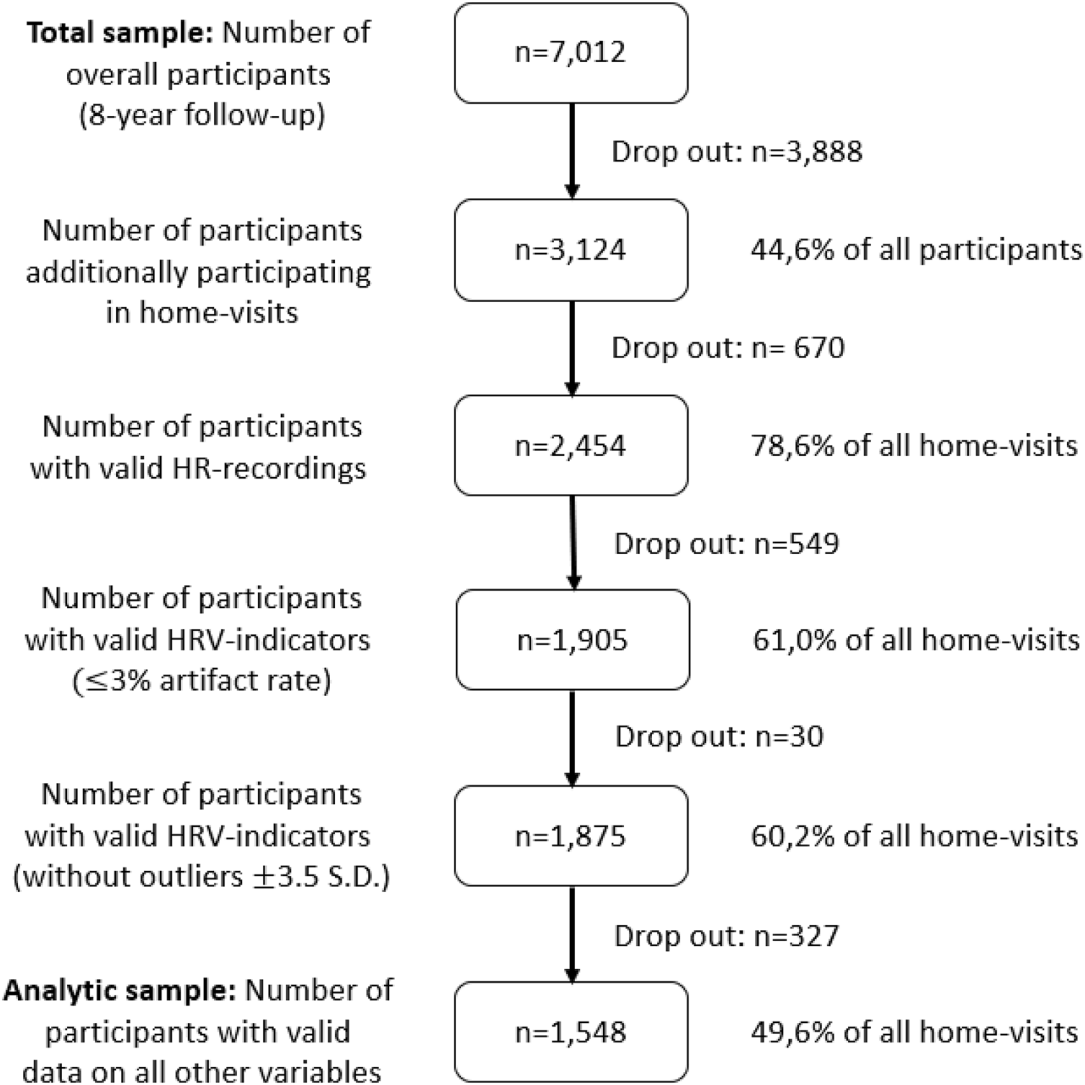
Flow diagram of analytical sample ***Note:*** HR = heart rate; HRV = heart rate variability; year = 2008–2010

We used cross-sectional data from the third (8-year) follow-up of participants in the ESTHER study (“Epidemiological Investigations of the Chances of Preventing, Recognizing Early and Optimally Treating Chronic Diseases in an Elderly Population”). Details about the study, its participants, and drop-out since baseline data collection have been published elsewhere (Lechner et al. 2016; Löw et al. 2004; Maatouk, 2016; Raum et al. 2007). The ESTHER study is an ongoing population-based cohort study of older adults living in the federal state of Saarland, Germany. General practitioners (GPs) recruited participants aged 50 to 75 years during a routine health check-up. In Germany, this check-up is offered every three years to all adults 35 years and older by their compulsory health insurances. In addition to age (50–75 years), insufficient language skills were considered an exclusion criterion for recruitment. Recruitment took place between July 2000 and December 2002. The cohort was followed up in intervals of 2–3 years. At the 8-year follow-up, between 2008 and 2010, a total of 7012 participants took part in the study (response rate = 60.9%). In a first step, participants were required to fill out a standardized questionnaire either by themselves or with the support of their GP. In a second step, the participants were invited to also take part in a home visit conducted by trained study physicians during which they collected, among others, participants’ heart rate (HR). Overall, 3124 (44.6%) of all participants agreed to participate in these home visits. Valid data on ANS function measured by HRV were obtained from 1905 participants (61.0% of all home visits). In the final analyses we included individuals who participated in the home visits and furthermore provided complete and valid information on dependent variables and most control variables described more fully below (*n* = 1548 [49.6% of all home visits]). A detailed overview on how we arrived at the analytical sample is shown in Fig. 1. The ESTHER study was conducted in concordance with the Declaration of Helsinki and approved by the Ethics Committees of the respective faculties and associations. Study participants provided written informed consent prior to examination.

### Data collection

Prior to home visits during the 8-year follow-up, all participants completed a self-administered questionnaire covering socio-demographic, lifestyle, and medical details. Study physicians obtained further data during home visits using a standardized form. During these home visits, participants were interviewed on a variety of topics (e.g., social ties, smoking status) and they performed several physical and mental tasks (see below) under the direction of the study physician. Study physicians measured height and body weight on site and attached the HR-recorders at the beginning of the visit and detached them at the end. Home visits lasted on average 2.3 (± 1.1) hours. Similar to previous research, during HRV measurement a protocol consisting of phases of stress and recovery was applied (e.g., Maunder et al. 2012). The protocol consisted of five phases: (1) baseline, (2) stress, (3) recovery, (4) stress, and (5) recovery. During the first stress phase, participants performed the Short Physical Performance Battery Test (SPPB), consisting of: tandem stand, walking over four meters, and sit-to-stand test. In the second stress phase, the Mini-Mental Status Examination (MMSE) was performed. The MMSE screens orientation, retention, attention and numeracy, memory ability, and language. In the recovery phases the participants answered the questionnaires.

### Measurements

#### Heart rate variability (HRV)

HRV is a well-recognized measure of ANS function and is defined as the variation in time intervals between consecutive heartbeats (Acharya et al. 2006). A dysregulated ANS is limited in its ability to increase or decrease HR to adapt to the social and environmental demands—this is reflected in lower HRV (Thayer et al. 2012). Higher HRV indicates greater adaptability to differing demands and unpredicted stimuli (Acharya et al. 2006; Thayer et al. 2012).

HR was recorded as raw beat-to beat intervals using an ambulatory HR-recorder (5-lead CardioScout M-Channel, PicoMed) sampling at 500 Hz. Beat-to-beat intervals are the interval between two successive R-spikes. Researchers at the Center for Neuropsychological Research (University of Trier, Germany) processed all HR recordings according to the Task Force Guidelines (Heart rate variability: standards of measurement, physiological interpretation and clinical use. Task Force of the European Society of Cardiology and the North American Society of Pacing and Electrophysiology 1996). They decomposed the length of the recordings into 5.35-min blocks and calculated HRV indicators and artifact rate per block using the NEUROCOR® ANS-Explorer V3.5.11 (Wittling 2017).

Calculated HRV indicators included frequency domain measures such as low frequency (LF-HRV; ms^2^; 0.04–0.15 Hz), very low frequency (VLF-HRV; ms^2^; 0.003–0.04 Hz), and high frequency (HF-HRV; ms^2^; 0.15–0.4 Hz), as well as time domain measures such as the standard deviation of all normal-to-normal intervals (SDNN) in milliseconds and the root mean square of successive differences (RMSSD) in milliseconds in all valid adjacent beat-to-beat intervals (Shaffer and Ginsberg 2017). LF-HRV, VLF-HRV, and SDNN reflect both sympathetic and parasympathetic activity, while HF-HRV and RMSSD reflect primarily parasympathetic activity (Thayer et al. 2012). Overall, we obtained HRV indicators from 2454 HR recordings (78.6% of all home visits). We excluded all blocks with more than a 3% artifact rate from the analysis (51.8% of all blocks) and then calculated an average value for all HRV indicators per participant across all remaining 5.35-min blocks. (Note that a less conservative exclusion criterion of 5% artifact rate leads to similar results in the regression analysis.) Additionally, we excluded extreme data points (outliers) outside a range of 3.5 standard deviations (SD) from the mean (*n* = 30, 1.6% of remaining respondents), which might distort the analysis.

#### Supportive Social Ties

To measure different types of supportive social ties we used the short version of the Lubben Social Network Scale (LSNS-6, (Lubben et al. 2006)). The LSNS-6 is a self-report measure that evaluates both supportive family and friendship ties and was validated in a sample of older community-dwelling adults (Lubben et al. 2006). The LSNS-6 consists of six items, three of which refer to supportive family ties with the remaining three items assessing supportive friendship ties. Items refer to the number of family members/friends, respondents see or hear from at least once a month, the number of family members/friends one feels close to such that one could call on them for help, and the number of family members/friends with whom one feels at ease with and could talk about private matters. Answer categories included 0 “none”, 1 “one”, 2 “two”, 3 “three or four”, 4 “five through eight”, and 5 “nine or more”. For the overall score, we calculated a sum of all six items, a higher score indicating more supportive ties. In line with Lubben et al. (2006) we used the same approach to calculate sub-scores for either supportive family or supportive friendship ties, each based on three corresponding items. To ensure comparability between the overall score and the two sub-scores, the overall score was transformed to fit the range of the two sub-scores, ranging from 0 to 15.

#### Covariates and potential mediators

We included data on socio-demographic characteristics known to be associated with ANS function such as gender (female vs. male), age, educational attainment, marital status, and living arrangements (Abhishekh et al. 2013; Randall et al. 2009). According to the statutory retirement age in Germany, age was dichotomized as ≤ 65 (below retirement age) vs. > 65 years (above retirement age). Educational attainment was measured using the International Standard Classification of Education (ISCED 2011, categories: primary education, lower secondary education, and upper secondary education, see UNESCO (2012)). Due to the small number of respondents reporting primary educational attainment, the categories primary educational and lower secondary educational attainment were combined into a single category (reference category). Marital status was defined as being either married (reference category), or not (single, widowed, or divorced). Living arrangements described whether the respondent lived alone (yes vs. no). Due to the epidemiological nature of this study, participants taking medication potentially affecting HRV (e.g., beta-blockers, ACE inhibitors, antiarrhythmics, and antidepressants (Loellgen 2011)) were not excluded, but a dichotomous indicator to control for their use (yes vs. no) was created.

We assessed body mass index (BMI = kg/m^2^), an indicator of risk of chronic disease or physical health (Telford 2007), and smoking status (never smoker, former smoker, smoker). BMI was categorized into three groups (WHO 2017): normal weight (18.5 ≤ BMI < 25.0; reference category), overweight (25.0 ≤ BMI < 30.0), and obesity (BMI ≥ 30.0). The few respondents with complete data and a BMI smaller than 18.5 (underweight; *n* = 5) were excluded. Physical fitness was measured using the validated short physical performance battery (SPPB; range 0–12), testing balance, strength, gait, and endurance in older adults (Guralnik 1994), with higher values indicating better physical fitness. Psychological and cognitive aspects of health included cognitive functioning and signs of depression. Cognitive functioning was measured using the validated Mini-Mental Status Examination (MMSE; range 1–30) (Folstein et al. 1975). The psychometric properties of the MMSE have been tested in a German primary care setting and were assessed to be satisfactory (Stein, 2015). The scale was categorized into “no cognitive impairment” (reference category; > 28), “mild cognitive impairment” (21–28), “moderate cognitive impairment” (11–20), “severe cognitive impairment” (1–10), and “not specified” (missing value). Due to the small number of respondents with symptoms of moderate cognitive impairment (*n* = 4), the categories “mild cognitive impairment” and “moderate cognitive impairment” were combined. Depressive symptoms were assessed using the Geriatric Depression Scale (GDS-15; range 0–15), a reliable and validated screening tool for depression among older adults (Yesavage and Sheikh 1986), also in German (Baumgartner et al. 2019). The total score was categorized into “no signs of depression” (reference category; 0–5), “signs of mild to moderate depression” (6–10), “signs of severe depression” (11–15), and “not specified” (missing value). Additional sensitivity analyses revealed that the results were similar albeit weaker after exclusion of respondents without valid date on cognitive functioning and depressive symptoms (data not shown).

### Statistical analysis

Descriptive statistics are presented by reporting means and standard deviations (SDs) for continuous variables and percentage distributions for categorical variables. Group differences in supportive social ties were tested using two-sample T-tests. Pearson's correlation coefficients were used to indicate the correlation between our indicators.

Internal consistency of the metric indices was reported using Cronbach’s alpha.

Due to their skewed distribution, all HRV indicators were logarithmically transformed. Multivariate linear regression analyses were performed to explore associations between supportive social ties (predictor variables) and HRV indicators (dependent variables). Separate regression analyses were performed, which included either the LSNS overall score or both sub-scores for family and friendship ties. Regression coefficients are presented as the percentage change in HRV indicators if the independent variables change by one unit. A test for multicollinearity was performed in models including all dependent and control variables.

A hierarchical modeling strategy was used. To differentiate between control variables and potential mediators (behavioral/physical and psychological/cognitive health indicators) as proposed by Uchino (2006), models were adjusted in several steps: Model 1 was adjusted for age, gender, and medication use; Model 2 included further adjustment for marital status and living arrangements; Model 3 included additional adjustment for weight categories, smoking status, and physical fitness; and the final model (Model 4) was also adjusted for measures of depression and cognitive functioning.

We additionally tested whether the association between supportive social ties was moderated across different subgroups according to their marital status (married vs. not married) or their age (below vs. above retirement age). Additionally, we performed three-way interactions between supportive social tie indicators, marital status, and age. Results from these analyses are presented as average marginal effects across subgroups, that is the slopes of the association between supportive social ties and HRV within the categories of marital status or age.

An alpha level of 0.1 in 2-sided testing was used to determine marginal statistical significance and an alpha level of 0.05 determined strong statistical significance. All data analyses were performed in STATA 13.1 (StataCorp 2013).

## Results

Descriptive statistics can be found in Table 1. Cronbach’s alphas for the scale measuring overall supportive social ties and the subscales measuring supportive family and friendship ties were 0.74, 0.79, and 0.68, respectively, suggesting mostly acceptable internal consistency (Sharma 2016). The average level of overall supportive ties (8.7; ± 2.6) was in the upper third of the LSNS (Table 1). On average, however, respondents reported more supportive family ties (9.6; ± 2.9) than those from friends (7.9; ± 23.3; [*p* ≤ 0.001]).

**Table 1:** Descriptive statistics of the study sample (*N* = 1548)

Variable		*n*	%	mean	SD
Gender					
Male*		708	45.7		
Female		840	54.3		
Age, years				68.7	6.1
Age (dichotomized)^j^					
Below statutory retirement age		503	32.5		
Above statutory retirement age		1,045	67.5		
Educational attainment^a^					
Upper secondary		262	16.9		
Lower secondary*	}combined for analysis	1,270	82.0		
Primary*		16	1.0		
Marital status					
Married*		1,134	73.3		
Single		55	3.6		
Divorced		108	7.0		
Widowed		251	16.2		
Living alone					
No*		1,253	80.9		
Yes		295	19.1		
Use of HRV-influencing medication					
No*		937	60.5		
Yes		611	39.5		
BMI^b^					
Normal weight* (18.5≤BMI<25.0)		384	24.8		
Overweight (25.0≤BMI<30.0)		673	43.5		
Obese (BMI≥30.0)		491	31.7		
Physical fitness^c^				10.0	1.7
Smoking status					
Non-smoker*		868	56.1		
Ex-smoker		577	37.3		
Smoker		103	6.7		
Cognitive functioning^d^					
No cognitive impairment*		836	54.0		
Mild cognitive impairment	}combined for analysis	568	36.7		
Moderate cognitive impairment		4	0.3		
Not specified		140	9.0		
Signs of depression^e^					
No signs of depression*		1,293	83.5		
Signs of mild to moderate depression		153	9.9		
Signs of severe depression		48	3.1		
Not specified		54	3.5		
*Independent variables*					
Supportive social ties^f^					
Overall supportive ties				8.7	2.6
Supportive family ties				9.6	2.9
Supportive friendship ties				7.9	3.3
*Dependent variables *					
HRV^g^ indicators					
Low frequency (LF)				370.9	276.1
Very low frequency (VLF)				763.2	602.4
High frequency (HF)				132.9	135.9
SDNN^h^				44.2	13.8
RMSSD^i^				26.1	10.4
*N*		1,548	100.0		

Year = 2008–2010; *reference category; ^a^measured using the International Standard Classification of Education (ISCED 2011); ^b^*BMI* Body Mass Index in kg/m^2^; ^c^measured using the Short Physical Performance Battery; ^d^measured using the Mini-Mental Status Examination assessing cognitive functioning; ^e^measured using the Geriatric Depression Scale higher values indicating signs of depression; ^f^measured using the Lubben Social Network Scale higher values indicating better physical fitness; ^g^*HRV* Heart rate variability; ^h^*SDNN* standard deviation of all normal-to-normal intervals; ^i^*RMSSD* root mean square of successive differences, ^j^according to the statutory retirement age in Germany

### Bivariate analyses

With a few exceptions, all indicators showed a small to moderate correlation (Supplementary Table S1). Among others, stronger correlations were found between the HRV indicators and between marital status and living alone (while 98.7% of all married individuals lived with at least one other person, only 32.4% of all individuals that were not married lived with at least one other person). Married individuals reported on average more supportive family ties (10.0; ± 2.7) than unmarried individuals (8.4; ± 3.3; *p* ≤ 0.001), while there were no differences in supportive friendship ties (married: 8.0, ± 3.3; unmarried: 7.7, ± 3.3; *p* = 0.104). Adults below retirement age reported significantly more supportive friendship ties (8.4; ± 3.2) than those above retirement age (7.7; ± 3.3; *p* ≤ 0.001), but there were no significant differences between supportive family ties (below: 9.7, 2.9; above: 9.5, ± 2.9; *p* = 0.195).

### Multivariate analyses

Tables 2 and 3 demonstrate associations between supportive social ties and various HRV indicators. A test for multicollinearity in model 4 including all dependent and control variables indicated moderate multicollinearity between all dependent and control variables (VIF = 0.03–2.35). We observed small but significant positive associations between supportive social ties and HRV indicators that reflect both sympathetic and parasympathetic activity (LF-HRV, VLF-HRV, SDNN), while indicators reflecting only parasympathetic activity showed close to no association (RMSSD, HF-HRV).

**Table 2:** Associations between overall supportive social ties and heart rate variability – results from linear regression analysis (*N* = 1548)

Social ties	LF-HRV				VLF-HRV				SDNN			
	M 1	M 2	M 3	M 4	M 1	M 2	M 3	M 4	M 1	M 2	M 3	M 4
Overall supportive ties	2.4*	1.9*	1.0	0.6	2.5**	2.0*	1.1	1.0	1.1**	0.9*	0.5^†^	0.5
*R*^*2*^	*0.082*	*0.090*	*0.151*	*0.156*	*0.041*	*0.049*	*0.124*	*0.127*	*0.036*	*0.044*	*0.121*	*0.124*
Supportive social ties in												
Married		1.4	0.6	0.2		1.8^*^	1.1	0.9		0.8*	0.5	0.4
Unmarried		3.0*	1.8	1.4		2.5^†^	1.2	1.1		1.2*	0.6	0.6
*R*^*2*^		*0.091*	*0.151*	*0.157*		*0.049*	*0.124*	*0.127*		*0.044*	*0.121*	*0.124*
Supportive social ties in respondents												
Below retirement age	2.7^†^	2.2	1.3	0.9	2.9*	2.4^†^	1.5	1.3	1.4*	1.2*	0.8	0.7
Above retirement age	2.2*	1.7^†^	0.8	0.5	2.4**	1.8*	0.9	0.8	1.0**	0.8*	0.4	0.4
*R*^*2*^	*0.082*	*0.090*	*0.151*	*0.156*	*0.041*	*0.049*	*0.125*	*0.127*	*0.036*	*0.044*	*0.121*	*0.124*

Social ties	RMSSD				HF-HRV			
	M 1	M 2	M 3	M 4	M 1	M 2	M 3	M 4
Overall supportive ties	0.6	0.4	0.3	0.2	1.3	0.9	0.4	0.3
*R*^*2*^	*0.004*	*0.008*	*0.022*	*0.023*	*0.012*	*0.019*	*0.039*	*0.040*
Supportive social ties in								
Married		0.5	0.3	0.3		0.7	0.3	0.2
Unmarried		0.3	0.1	0.1		1.4	0.7	0.7
*R*^*2*^		*0.008*	*0.022*	*0.023*		*0.019*	*0.039*	*0.040*
Supportive social ties in respondents								
Below retirement age	0.8	0.6	0.5	0.4	1.9	1.5	1.0	0.9
Above retirement age	0.5	0.3	0.2	0.2	1.0	0.6	0.1	0.0
*R*^*2*^	*0.004*	*0.008*	*0.022*	*0.023*	*0.012*	*0.019*	*0.039*	*0.040*

^†^*p* < .1; *p < .05; **p < .01; year = 2008–2010; regression coefficients represent changes in %; all interaction terms are presented as average marginal effects, i.e., the slopes for the social support indicators by marital status or age group; *HRV *heart rate variability; *LF* low frequency; *VLF* very low frequency; *HF* high frequency; *RMSSD* root mean square of successive differences; SDNN = standard deviation of all normal-to-normal intervals; Model 1 (M1) controlled for retirement age (≤ 65 vs. > 65 years), gender, educational attainment, use of HRV-influencing medication; Model 2 (M2) also controlled for marital status (married vs. unmarried) and living alone; Model 3 (M3) also controlled for obesity/overweight and smoking status; Model 4 (M4) also controlled for signs of cognitive impairment and depression; Models 1 and 2 were condensed into a single model (2) if they include an interaction term with marital status

**Table 3:** Associations between supportive family and friendship ties and heart rate variability–results from linear regression analysis (*N* = 1548)

Social ties	LF-HRV				VLF-HRV				SDNN			
	M 1	M 2	M 3	M 4	M 1	M 2	M 3	M 4	M 1	M 2	M 3	M 4
Supportive family ties	1.0	0.3	0.2	0.1	1.1	0.4	0.3	0.3	0.4	0.1	0.1	0.1
Supportive friendship ties	1.3^*^	1.4*	0.7	0.5	1.4*	1.5*	0.7	0.6	0.7*	0.7*	0.4	0.3
*R*^*2*^	*0.082*	*0.091*	*0.151*	*0.156*	*0.041*	*0.050*	*0.125*	*0.127*	*0.036*	*0.045*	*0.121*	*0.124*
Supportive family ties in												
Married		0.7	0.8	0.7		0.7	0.9	0.8		0.3	0.3	0.3
Unmarried		− 0.5	− 0.8	− 0.9		− 0.2	− 0.6	− 0.7		− 0.1	− 0.2	− 0.3
Supportive friendship ties in												
Married		0.7	− 0.1	− 0.3		1.0	0.3	0.2		0.5^†^	0.2	0.1
Unmarried		3.5**	2.5*	2.5*		2.7*	1.8	1.7		1.2*	0.8^†^	0.8^†^
*R*^*2*^		*0.093*	*0.153*	*0.159*		*0.051*	*0.125*	*0.129*		*0.046*	*0.122*	*0.125*
Supportive family ties in respondents												
Below retirement age	2.9*	2.2^†^	2.2^†^	2.1^†^	1.7	1.0	1.0	1.0	0.9^†^	0.6	0.7	0.6
Above retirement age	0.1	− 0.6	− 0.7	− 0.8	0.8	0.1	0.0	− 0.0	0.2	− 0.1	− 0.1	− 0.1
Supportive friendship ties												
Below retirement age	− 0.0	0.1	− 0.7	− 0.9	1.2	1.3	0.5	0.3	0.5	0.5	0.2	0.1
Above retirement age	1.9*	2.1**	1.3^†^	1.1	1.5^*^	1.6^*^	0.8	0.8	0.8*	0.8*	0.5	0.4
*R*^*2*^	*0.085*	*0.094*	*0.153*	*0.159*	*0.041*	*0.050*	*0.125*	*0.128*	*0.037*	*0.046*	*0.122*	*0.125*

Social ties	RMSSD				HF-HRV			
	M 1	M 2	M 3	M 4	M 1	M 2	M 3	M 4
Supportive family ties	0.2	− 0.0	− 0.0	− 0.0	0.2	− 0.4	− 0.4	− 0.5
Supportive friendship ties	0.4	0.4	0.3	0.2	1.0	1.1^†^	0.7	0.7
*R*^*2*^	*0.004*	*0.008*	*0.022*	*0.023*	*0.012*	*0.020*	*0.039*	*0.040*
Supportive family ties in								
Married		0.1	0.1	0.1		− 0.1	− 0.1	− 0.2
Unmarried		− 0.2	− 0.3	− 0.3		− 0.8	− 1.0	− 1.0
Supportive friendship ties in								
Married		0.3	0.2	0.2		0.7	0.4	0.3
Unmarried		0.5	0.3	0.3		2.1^†^	1.7	1.7
*R*^*2*^		*0.008*	*0.022*	*0.024*		*0.021*	*0.040*	*0.041*
Supportive family ties in respondents								
Below retirement age	0.7	0.5	0.5	0.5	1.7	1.2	1.2	1.0
Above retirement age	− 0.1	− 0.2	− 0.3	− 0.3	− 0.5	− 1.1	− 1.2	− 1.2
Supportive friendship ties in respondents								
Below retirement age	0.1	0.2	− 0.0	− 0.0	0.3	0.4	− 0.1	− 0.1
Above retirement age	0.5	0.5	0.4	0.4	1.3^†^	1.4^†^	1.1	1.0
*R*^*2*^	*0.005*	*0.009*	*0.023*	*0.024*	*0.014*	*0.022*	*0.041*	*0.042*

^†^*p* < .1; *p < .05; **p < .01; year = 2008–2010; regression coefficients represent changes in %; all interaction terms are presented as average marginal effects, i.e., the slopes for the social support indicators by marital status or age group; *HRV* heart rate variability; *LF* low frequency; *VLF* very low frequency; *HF* high frequency; *RMSSD* root mean square of successive differences; SDNN = standard deviation of all normal-to-normal intervals; Model 1 (M1) controlled for retirement age (≤ 65 vs. > 65 years), gender, educational attainment, use of HRV-influencing medication; Model 2 (M2) also controlled for marital status (married vs. unmarried) and living alone; Model 3 (M3) also controlled for obesity/overweight and smoking status; Model 4 (M4) also controlled for signs of cognitive impairment and depression; Models 1 and 2 were condensed into a single model (2) if they include an interaction term with marital status

Higher overall supportive social ties were associated with greater HRV (Table 2), as evidenced in Models 1 and 2, which control for socio-demographic characteristics, medication use, marital status, and living alone (LF-HRV, VLF-HRV, SDNN). In Models differentiating between family and friendship ties (Table 3), significant positive associations, albeit smaller in strength compared to the above-described associations with overall social ties, were present only for supportive friendship ties and HRV in the first two Models (LF-HRV, VLF-HRV, SDNN). Positive associations of all social tie indicators were noticeably reduced in size and significance, after controlling for behavioral/physical and psychological/cognitive indicators.

Tables 2 and 3 further show the slopes of the association between supportive social ties and HRV indicators within different subgroups based on marital status or age. Supportive friendship ties showed significant positive associations in individuals that were either unmarried (LF-HRV, VLF-HRV, SDNN) or above retirement age (LF-HRV, VLF-HRV, SDNN). The former association furthermore remained mostly significant even after including both behavioral and psychological indicators (LF-HRV). Supportive family ties showed significant associations in individuals below retirement age (LF-HRV, SDNN).

Three-way interactions between overall supportive ties, marital status, and age showed significant positive associations in individuals that were both unmarried and above retirement age (Model 2: 1.3–4.0%; LF, VLF, SDRR; *p* ≤ 0.1) and married individuals below retirement age (Model 2; 1.5–3.3%; LF, VLF, SDRR; *p* ≤ 0.1) before controlling for behavioral/physical and psychological/cognitive indicators. No significant associations were found in models of RMSSD and HF-HRV, as well as in models controlling for behavioral/physical and psychological/cognitive indicators. In models differentiating the source of supportive ties, family ties showed positive associations in married individuals below retirement age (Model 2: 1.2–3.8%; LF, SDRR, HF; *p* ≤ 0.1), even after controlling for behavioral/physical and psychological/cognitive indicators (Model 4: 1.2–3.4%; LF, SDRR; *p* ≤ 0.1). Friendship ties showed positive associations in unmarried individuals above retirement age (Model 2: 1.4–4.9%; LF, VLF, SDRR; *p* ≤ 0.1), even after controlling for behavioral/physical and psychological/cognitive indicators (Model 4: 1.1–4.1%; LF, VLF, SDRR; *p* ≤ 0.1).

## Discussion

In this study, we explore associations between supportive social ties and ANS function, a physiological indicator of health, in a sample of older adults living in Germany. Previous studies on social ties and ANS function as measured by HRV have reported positive associations (Gouin et al. 2015; Hemingway et al. 2005; Horsten et al. 1999) with one study finding no significant associations (Britton et al. 2007). The present study extends this line of inquiry as it (a) focuses on the association between different sources of social support and ANS function and (b) explores these associations in different life circumstances (based on marital status and age).

Our results overall suggest that supportive social ties, especially friendship ties, are positively associated with better ANS function. Significant associations of supportive social ties were generally diminished or disappeared entirely after controlling for behavioral and psychological health indicators. Based on the model proposed by Uchino (2006) we expected such findings, as behavioral and psychological processes are thought to act as mediators between social ties and physiological processes.

Because family members are essential in providing instrumental support (Messeri et al. 1993), we would have expected positive associations, especially in individuals above retirement age or without a spouse. However, older adults may prefer to be independent and needing instrumental support may lead to emotional distress and feelings of vulnerability, thus diminishing the positive effects of support (Li and Zhang 2015). In line with this argumentation, Merz and Huxhold (2010) showed that instrumental support from family members increased negative affect, especially if the relationship quality is bad, while instrumental support from friends decreased negative affect.

By exploring different life circumstances, we observed that supportive friendship ties rather than family ties are associated with better ANS function in unmarried individuals and in respondents above the statutory retirement age. However, in respondents younger than the statutory retirement age, we found significant associations of supportive family ties. In married individuals we found no significant associations of friendship ties. In line with previous studies (Li et al. 2014) as well as theoretical considerations of both the hierarchical compensatory model and the task-specific model, these findings indicate that a spouse is the most important social tie. The number of further potential sources of support (friendships) may thus be less important if the spouse is able to provide sufficient levels of support firsthand. Even though we were not able to capture marital transitions, our finding that supportive friendship ties are most beneficial in unmarried individuals is similar to a study reporting that having a friend as a confidante was associated with better health after widowhood than having a family member as a confidante (Bookwala et al. 2014). Furthermore, another study indicated that while children showed increased support for a widowed parent directly after the loss of a spouse, increased support from friendship ties was observed for an even later period (Ha 2008). As discussed above, both friends and spouses are chosen based on similar interests and mutual affection and they play an important role in companionship. Thus, in cases of absence or loss of a spouse, friendship ties, especially in larger numbers, may serve as an alternative key source of support or companionship, which would also be in line with arguments from the task-specific model (Li et al. 2014).

Our results indicating that friendship ties seem to become more important in later years are consistent with another study focusing on well-being (Huxhold et al. 2013). However, our data also show that individuals above retirement age have on average fewer friendship ties. Based on both socio-emotional selectivity theory (Carstensen et al. 1999) and the strength and vulnerability model (Charles 2010), it is assumed that older individuals reduce negative social ties and focus on higher quality ties. Negative friendship ties are easier to terminate than family ties. In this case, the association found might also imply that a lower number of friends in older age can be as supportive as a higher number of friends in the lower age group because they might be higher quality relationships. Future studies should explore this in more detail.

Our overall findings related to friendship ties are in line with a previous study on associations between contact with ties and health (Thanakwang 2009), but they stand in contrast to others, which found that family ties rather than friendship ties were more important in their positive association with mortality and levels of inflammation markers (Shor et al. 2013; Uchino et al. 2015). The results of the present study, however, suggest that these associations may additionally depend on whether people are above or below retirement age. Thanakwang (2009), for example, studied a sample with a mean age of 69 years (±7.2) and 68% of the sample not being employed. Based on the assumption that a large part of this sample might be retired, their findings support our results in individuals above the statutory retirement age. While Shor et al. (2013) did not give an exact mean age of the sample, they did mention that especially age groups above the age of 40 were represented. The study sample of Uchino et al. (2015) had a mean age of 42.4 years, and a younger sample might explain why their findings seem to be contrary to those of Thanakwang (2009). However, we must note that differences are also likely to be based on the various cultural contexts of the studies.

The results showed some differences between the types of indicators of ANS function. While we found results that were mostly significant for indicators of ANS function known to reflect both sympathetic and parasympathetic activity (LF-HRV, VLF-HRV, and SDNN), we did not observe similar findings for RMSSD and HF-HRV, indicators reflecting primarily parasympathetic activity. This is in line with the study of Horsten et al. (1999). Other studies have also found significant associations between social support (Randall et al. 2009) or marital status (Hemingway et al. 2005) and at least two of these former three HRV indicators. However, in contrast to some studies, we could find fewer and weaker associations between social support and HF-HRV (Gouin et al. 2015; Hemingway et al. 2005). Missing significant associations using indicators of primarily parasympathetic activity in the present analysis might be explained by age as parasympathetic activity decrease over the lifespan and differences become smaller and more difficult to detect. (Note that both studies finding significant associations for HR-HRV used younger study samples with a mean age of 23.8 years (±3.5) in the study of Gouin et al. (2015) and a range of 45–68 years in the study of Hemingway et al. (2005).)

### Strengths and limitations

A strength of this study is the use of HRV, an objective, non-invasive measure of ANS function. HRV also has the benefit of recognition as an intermediate health outcome of its own (Acharya et al. 2006; Thoits 2011) that is responsive to intervention (Gademan 2007), and therefore has value as a potential measure of effectiveness in future intervention studies.

Limitations of the present study are that HRV was assessed at only one time point and based on a cross-sectional design causality cannot be established nor can we exclude reverse causation. For example, individuals that are frail or sick might not be able to foster their relationships and thus become more isolated. Additionally, it has been shown that older people tend to reduce their weaker non-kin ties if their physical health status declines (Cornwell 2009). This may be an alternative explanation to our results based on age. However, intimate friendships are often preserved, especially if they are beneficial (Li and Zhang 2015). The few longitudinal studies analyzing overall social ties and ANS function (Britton et al. 2007; Gouin et al. 2015) found mixed results (either none or positive associations). There is therefore still need for longitudinal studies that differentiate between type of social ties and observe life transitions (e.g., retirement). Another limitation is a possible selection bias with healthier ESTHER participants taking part in the follow-ups and home visits. Furthermore, there might be a missing values bias, as half of the sample was excluded based on invalid or missing data.

Our findings regarding marital status are furthermore limited because we were not able to differentiate between unmarried individuals with and without a non-marital partner. It could be argued that the support of a non-marital partner, especially a cohabiting partner, is similar to a wedded spouse as older adults might seek cohabitation as an alternative to marriage (Brown et al. 2006). One study, for example, did not find differences in psychological well-being based on being married or cohabitating with a partner and even suggested a more prominent cohabitation advantage than marriage advantage in men (Wright and Brown 2017). Another study indicates that even though cohabiting partners are less likely to offer partner care than their married counterparts, the quantity of support is similar to married partners once support is offered (Noël-Miller 2011). The differences might therefore be larger if comparing married individuals to unmarried individuals without a non-marital partner and smaller if comparing married individuals to unmarried individuals with a non-marital/cohabiting partner.

Additionally, the LSNS-6 does neither differentiate between a partner and other family ties as well as social ties within and outside the household. Future studies might add to our study by further differentiating between different social ties. For example, while having a spouse or partner is often associated with better mental and physical health, having (coresident) children shows rather mixed results (Grundy et al. 2019; Mair 2013).

Our indicator for supportive social ties is also limited, because it does not differentiate between type of support (instrumental, emotional, etc.). For example, a study differentiating sources of support reported that instrumental support was positively associated with well-being when received from non-kin and negatively associated when instrumental support was provided by their family ties (Merz and Huxhold 2010). This study also reported positive associations of emotional support from family ties but not from non-kin (Merz and Huxhold 2010).

In addition, our measure of social ties does not account for negative or potentially deleterious relationships. Previous studies suggest, for example, that negative relationships are a risk factor for health (Finch et al. 1989) and that friendship ties become more important if the marriage quality is bad (Han et al. 2019). The exclusion of such negative ties would mean that we underestimate the association between supportive ties and ANS function (Holt-Lunstad and Smith 2012).

### Conclusions

This study provides evidence that supportive family and friendship ties have different associations with ANS function based on certain life circumstances. Our descriptive results show that individuals who are unmarried or are above retirement age report on average fewer supportive social ties and could therefore be more vulnerable for social isolation. We also observed that friendship ties in particular contribute to better health in these vulnerable subgroups. Moreover, we conclude that programs supporting the development or maintenance of friendship ties might be especially beneficial for these subgroups. Our results are important especially in view of demographic change, specifically, the aging of the population. Thus, our findings can be helpful in designing targeted intervention strategies for unmarried individuals and individuals above retirement age to help them retain established friendships and generate new social connections. A recent review has found that the quality of interventions reducing social isolation or loneliness in older adults is rather weak, but common characteristics of interventions providing evidence for decreased social isolation were, for example, being able to adapt the intervention to the local target group, involving the target group in the process, or providing productive activities that bring people together (Gardiner et al. 2018). Future studies might take these results into account to develop interventions that specifically create more possibilities for older adults to establish and create friendship ties.

## Supplementary Information

Below is the link to the electronic supplementary material.

Supplementary file1 (DOCX 22 kb)

## Data Availability

Due to the sensitive nature of the medical examinations and the questions asked in this study, survey respondents were assured that raw data would remain confidential and would not be shared.

## Code availability

All analyses were conducted using STATA, and the code is available on request.
