# Supplementary material for: The association between supportive social ties and autonomic nervous system function—differences between family ties and friendship ties in a cohort of older adults
Source: Eur J Ageing. 2021 Jul 9;19(2):263–76. doi: 10.1007/s10433-021-00638-2 (PMC9156582; doi:10.1007/s10433-021-00638-2)
Supplement: Supplementary file 1 — Supplementary file1 (DOCX 22 kb) [file 10433_2021_638_MOESM1_ESM.docx]

**Supplementary Table S1:** Correlations (*r*) and p-values (*p*) of main variables in the study

|  |  | Female | Age (years) | Being above retirement age | Educational attainment | Being unmarried | Living alone | Use of HRV-influencing medication |
| --- | --- | --- | --- | --- | --- | --- | --- | --- |
| Age (years) | *r* | -0.0945 | 1 |  |  |  |  |  |
|  | *p* | 0.0002 |  |  |  |  |  |  |
| Being above retirement age | *r* | -0.0832 | 0.8053 | 1 |  |  |  |  |
|  | *p* | 0.0010 | <0.0001 |  |  |  |  |  |
| Educational attainment | *r* | -0.1908 | -0.0184 | -0.0032 | 1 |  |  |  |
|  | *p* | <0.0001 | 0.4695 | 0.9002 |  |  |  |  |
| Being unmarried | *r* | 0.2647 | 0.1204 | 0.0421 | -0.0314 | 1 |  |  |
|  | *p* | <0.0001 | <0.0001 | 0.0974 | 0.2168 |  |  |  |
| Living alone | *r* | 0.2573 | 0.1614 | 0.0978 | -0.0085 | 0.7473 | 1 |  |
|  | *p* | <0.0001 | <0.0001 | 0.0001 | 0.7394 | <0.0001 |  |  |
| Use of HRV-influencing medication | *r* | -0.0572 | 0.1462 | 0.1003 | -0.0755 | 0.0077 | -0.0048 | 1 |
|  | *p* | 0.0245 | <0.0001 | 0.0001 | 0.0030 | 0.7608 | 0.8492 |  |
| Obesity | *r* | -0.1040 | 0.0019 | -0.0152 | -0.0233 | -0.0363 | -0.0492 | 0.1867 |
|  | *p* | <0.0001 | 0.9394 | 0.5510 | 0.3603 | 0.1535 | 0.0530 | <0.0001 |
| Physical fitness | *r* | -0.0807 | -0.2468 | -0.1544 | 0.1011 | -0.1131 | -0.1043 | -0.1487 |
|  | *p* | 0.0015 | <0.0001 | <0.0001 | 0.0001 | <0.0001 | <0.0001 | <0.0001 |
| Ex-smoker | *r* | -0.2524 | -0.0423 | -0.0186 | 0.0155 | -0.0824 | -0.0611 | 0.0444 |
|  | *p* | <0.0001 | 0.0959 | 0.4652 | 0.5430 | 0.0012 | 0.0162 | 0.0805 |
| Smoker | *r* | -0.0202 | -0.1314 | -0.1136 | -0.0030 | 0.0202 | 0.0157 | 0.0177 |
|  | *p* | 0.4260 | <0.0001 | <0.0001 | 0.9064 | 0.4265 | 0.5383 | 0.4855 |
| Cognitive functioning | *r* | 0.0602 | -0.1896 | -0.1350 | 0.1533 | 0.0451 | 0.0244 | -0.0873 |
|  | *p* | 0.0239 | <0.0001 | <0.0001 | <0.0001 | 0.0904 | 0.3608 | 0.0010 |
| Signs of depression | *r* | 0.0872 | -0.0733 | -0.0872 | -0.0676 | 0.1335 | 0.1134 | 0.1274 |
|  | *p* | 0.0012 | 0.0066 | 0.0012 | 0.0122 | <0.0001 | <0.0001 | <0.0001 |
| Overall supportive ties | *r* | 0.0284 | -0.0938 | -0.0870 | 0.0576 | -0.1601 | -0.1592 | -0.0821 |
|  | *p* | 0.2640 | 0.0002 | 0.0006 | 0.0235 | <0.0001 | <0.0001 | 0.0012 |
| Supportive family ties | *r* | 0.0226 | -0.0347 | -0.0329 | 0.0118 | -0.2328 | -0.2300 | -0.0592 |
|  | *p* | 0.3751 | 0.1728 | 0.1953 | 0.6431 | <0.0001 | <0.0001 | 0.0198 |
| Supportive friendship ties | *r* | 0.0239 | -0.1140 | -0.1050 | 0.0783 | -0.0413 | -0.0424 | -0.0743 |
|  | *p* | 0.3480 | <0.0001 | <0.0001 | 0.0021 | 0.1041 | 0.0954 | 0.0035 |
| Very low frequency (VLF) | *r* | -0.0906 | -0.0977 | -0.0597 | 0.0085 | -0.1049 | -0.0975 | -0.0923 |
|  | *p* | 0.0004 | 0.0001 | 0.0189 | 0.7369 | <0.0001 | 0.0001 | 0.0003 |
| Low frequency (LF) | *r* | 0.0178 | -0.2630 | -0.2219 | 0.0259 | -0.0768 | -0.0724 | -0.1229 |
|  | *p* | 0.4844 | <0.0001 | <0.0001 | 0.3087 | 0.0025 | 0.0044 | <0.0001 |
| High frequency (HF) | *r* | 0.0665 | -0.0060 | -0.0274 | 0.0186 | -0.0033 | -0.0258 | 0.0766 |
|  | *p* | 0.0089 | 0.8150 | 0.2806 | 0.4642 | 0.8956 | 0.3095 | 0.0026 |
| LF/HF | *r* | -0.0745 | -0.2263 | -0.1634 | 0.0182 | -0.0578 | -0.0284 | -0.1892 |
|  | *p* | 0.0034 | <0.0001 | <0.0001 | 0.4736 | 0.0229 | 0.2636 | <0.0001 |
| SDNN | *r* | -0.0519 | -0.1464 | -0.1126 | -0.0039 | -0.1054 | -0.1120 | -0.0971 |
|  | *p* | 0.0411 | <0.0001 | <0.0001 | 0.8776 | <0.0001 | <0.0001 | 0.0001 |
| RMSSD | *r* | 0.0130 | 0.0308 | 0.0081 | 0.0248 | -0.0141 | -0.0398 | 0.0534 |
|  | *p* | 0.6102 | 0.2258 | 0.7504 | 0.3304 | 0.5793 | 0.1171 | 0.0355 |

**Supplementary Table S1:** continued

|  |  | Obesity | Physical fitness | Ex-smoker | Smoker | Cognitive functioning | Signs of depression | Overall supportive ties |
| --- | --- | --- | --- | --- | --- | --- | --- | --- |
| Age (years) | *r* |  |  |  |  |  |  |  |
|  | *p* |  |  |  |  |  |  |  |
| Being above retirement age | *r* |  |  |  |  |  |  |  |
|  | *p* |  |  |  |  |  |  |  |
| Educational attainment | *r* |  |  |  |  |  |  |  |
|  | *p* |  |  |  |  |  |  |  |
| Being unmarried | *r* |  |  |  |  |  |  |  |
|  | *p* |  |  |  |  |  |  |  |
| Living alone | *r* |  |  |  |  |  |  |  |
|  | *p* |  |  |  |  |  |  |  |
| Use of HRV-influencing medication | *r* |  |  |  |  |  |  |  |
|  | *p* |  |  |  |  |  |  |  |
| Obesity | *r* | 1 |  |  |  |  |  |  |
|  | *p* |  |  |  |  |  |  |  |
| Physical fitness | *r* | -0.1656 | 1 |  |  |  |  |  |
|  | *p* | <0.0001 |  |  |  |  |  |  |
| Ex-smoker | *r* | 0.1073 | 0.0578 | 1 |  |  |  |  |
|  | *p* | <0.0001 | 0.0230 |  |  |  |  |  |
| Smoker | *r* | -0.1181 | 0.0152 | -0.2058 | 1 |  |  |  |
|  | *p* | <0.0001 | 0.5500 | <0.0001 |  |  |  |  |
| Cognitive functioning | *r* | -0.0721 | 0.2242 | 0.0346 | -0.0031 | 1 |  |  |
|  | *p* | 0.0068 | <0.0001 | 0.1945 | 0.9088 |  |  |  |
| Signs of depression | *r* | 0.0564 | -0.1945 | -0.0109 | 0.0690 | -0.0372 | 1 |  |
|  | *p* | 0.0366 | <0.0001 | 0.6865 | 0.0105 | 0.1888 |  |  |
| Overall supportive ties | *r* | -0.0161 | 0.1851 | 0.0002 | -0.0662 | 0.1437 | -0.2471 | 1 |
|  | *p* | 0.5267 | <0.0001 | 0.9924 | 0.0091 | <0.0001 | <0.0001 |  |
| Supportive family ties | *r* | 0.0063 | 0.0923 | -0.0260 | -0.0617 | 0.0690 | -0.2022 | 0.7917 |
|  | *p* | 0.8038 | 0.0003 | 0.3070 | 0.0151 | 0.0096 | <0.0001 | <0.0001 |
| Supportive friendship ties | *r* | -0.0304 | 0.2038 | 0.0233 | -0.0476 | 0.1599 | -0.2005 | 0.8424 |
|  | *p* | 0.2322 | <0.0001 | 0.3599 | 0.0612 | <0.0001 | <0.0001 | <0.0001 |
| Very low frequency (VLF) | *r* | -0.1570 | 0.1644 | 0.0363 | -0.0818 | 0.0530 | -0.0194 | 0.0575 |
|  | *p* | <0.0001 | <0.0001 | 0.1538 | 0.0013 | 0.0469 | 0.4734 | 0.0237 |
| Low frequency (LF) | *r* | -0.1640 | 0.1782 | -0.0127 | -0.0576 | 0.1361 | -0.0468 | 0.0711 |
|  | *p* | <0.0001 | <0.0001 | 0.6175 | 0.0234 | <0.0001 | 0.0832 | 0.0051 |
| High frequency (HF) | *r* | -0.0353 | 0.0236 | -0.0318 | -0.0467 | 0.0686 | 0.0077 | 0.0140 |
|  | *p* | 0.1646 | 0.3540 | 0.2106 | 0.0663 | 0.0101 | 0.7764 | 0.5831 |
| LF/HF | *r* | -0.0781 | 0.1522 | 0.0583 | -0.0014 | 0.0830 | -0.0784 | 0.0578 |
|  | *p* | 0.0021 | <0.0001 | 0.0217 | 0.9556 | 0.0018 | 0.0036 | 0.0229 |
| SDNN | *r* | -0.2030 | 0.2071 | 0.0241 | -0.0770 | 0.0961 | -0.0691 | 0.0953 |
|  | *p* | <0.0001 | <0.0001 | 0.3428 | 0.0024 | 0.0003 | 0.0104 | 0.0002 |
| RMSSD | *r* | -0.0523 | 0.0399 | -0.0239 | -0.0458 | 0.0691 | -0.0031 | 0.0311 |
|  | *p* | 0.0397 | 0.1169 | 0.3476 | 0.0718 | 0.0094 | 0.9079 | 0.2215 |

**Supplementary Table S1:** continued

|  |  | Supportive family ties | Supportive friendship ties | Very low frequency (VLF) | Low frequency (LF) | High frequency (HF) | LF/HF | SDNN |
| --- | --- | --- | --- | --- | --- | --- | --- | --- |
| Age (years) |  |  |  |  |  |  |  |  |
|  |  |  |  |  |  |  |  |  |
| Being above retirement age |  |  |  |  |  |  |  |  |
|  |  |  |  |  |  |  |  |  |
| Educational attainment |  |  |  |  |  |  |  |  |
|  |  |  |  |  |  |  |  |  |
| Being unmarried |  |  |  |  |  |  |  |  |
|  |  |  |  |  |  |  |  |  |
| Living alone |  |  |  |  |  |  |  |  |
|  |  |  |  |  |  |  |  |  |
| Use of HRV-influencing medication | | |  |  |  |  |  |  |
|  |  |  |  |  |  |  |  |  |
| Obesity |  |  |  |  |  |  |  |  |
|  |  |  |  |  |  |  |  |  |
| Physical fitness |  |  |  |  |  |  |  |  |
|  |  |  |  |  |  |  |  |  |
| Ex-smoker |  |  |  |  |  |  |  |  |
|  |  |  |  |  |  |  |  |  |
| Smoker |  |  |  |  |  |  |  |  |
|  |  |  |  |  |  |  |  |  |
| Cognitive functioning |  |  |  |  |  |  |  |  |
|  |  |  |  |  |  |  |  |  |
| Signs of depression |  |  |  |  |  |  |  |  |
|  |  |  |  |  |  |  |  |  |
| Overall supportive ties |  |  |  |  |  |  |  |  |
|  |  |  |  |  |  |  |  |  |
| Supportive family ties |  | 1 |  |  |  |  |  |  |
|  |  |  |  |  |  |  |  |  |
| Supportive friendship ties | | 0.3378 | 1 |  |  |  |  |  |
|  |  | <0.0001 |  |  |  |  |  |  |
| Very low frequency (VLF) | | 0.0436 | 0.0501 | 1 |  |  |  |  |
|  |  | 0.0865 | 0.0486 |  |  |  |  |  |
| Low frequency (LF) |  | 0.0525 | 0.0633 | 0.5973 | 1 |  |  |  |
|  |  | 0.0389 | 0.0128 | <0.0001 |  |  |  |  |
| High frequency (HF) |  | 0.0110 | 0.0118 | 0.3170 | 0.5175 | 1 |  |  |
|  |  | 0.6650 | 0.6429 | <0.0001 | <0.0001 |  |  |  |
| LF/HF |  | 0.0561 | 0.0396 | 0.1508 | 0.3131 | -0.3325 | 1 |  |
|  |  | 0.0273 | 0.1191 | <0.0001 | <0.0001 | <0.0001 |  |  |
| SDNN |  | 0.0656 | 0.0890 | 0.8362 | 0.7886 | 0.5403 | 0.1348 | 1 |
|  |  | 0.0098 | 0.0005 | <0.0001 | <0.0001 | <0.0001 | <0.0001 |  |
| RMSSD |  | 0.0207 | 0.0296 | 0.3231 | 0.4649 | 0.8594 | -0.3983 | 0.5662 |
|  |  | 0.4149 | 0.2442 | <0.0001 | <0.0001 | <0.0001 | <0.0001 | <0.0001 |
